# Supplementary material for: APOE4 Drives Uniquely Dysfunctional Human Microglial States in Alzheimer’s Disease
Source: bioRxiv. 2026 Jun 19:2026.06.18.733295. Preprint. [Version 1] doi: 10.64898/2026.06.18.733295 (PMC13308055; doi:10.64898/2026.06.18.733295)
Supplement: Supplement 1 [file media-1.pdf]

Supplementary Figure 1

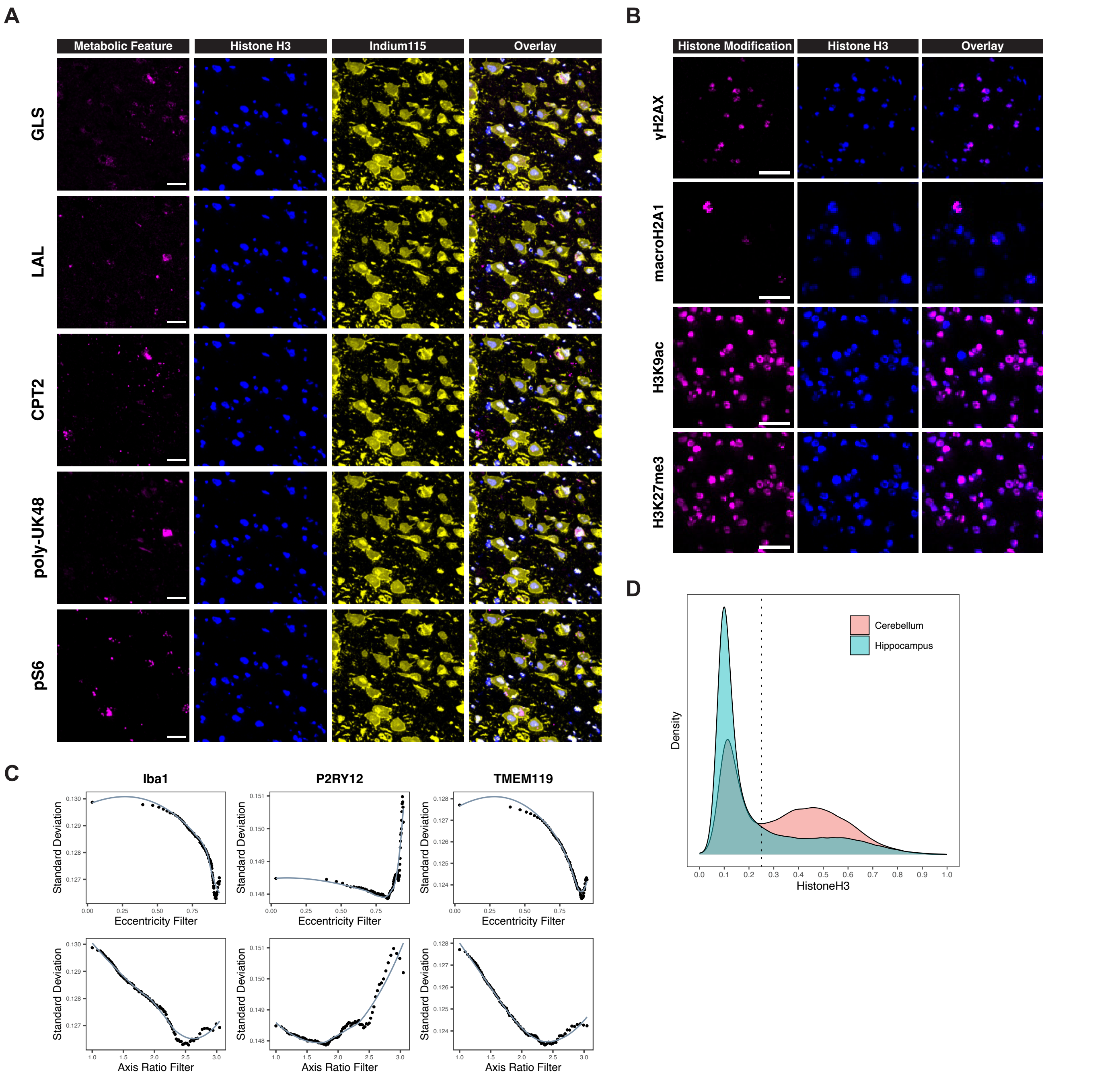

(A) Representative MIBI image demonstrating cytoplasmic localization of metabolic proteins within pyramidal neurons. Scale bars in multi-panel single-cell callout images (bottom) represent 10um. **(B)** Representative MIBI image demonstrating nuclear localization of histone modification markers. Scale bars in multi-panel single-cell callout images (bottom) represent 10um. **(C)** To refine morphological gating of anucleated microglia and exclude small transverse sections of glial processes, we systematically applied percentile-based morpho-filters across eccentricity and major-to-minor axis ratio. Specifically, for each percentile threshold n in the range 1-99, cells below the nth percentile for each parameter were progressively excluded. At each filtering step, we quantified the standard deviation (SD) of canonical microglial markers (Iba1, P2RY12, TMEM119) to assess marker stability under increasing stringency. The percentile cutoff at which the SD of marker expression reached a global minimum was identified as the optimal morphological filter. Based on these results, we retained only anucleated microglia that satisfied the following criteria: eccentricity ≥ 0.85 and major-to-minor

axis ratio ≥ 2. **(D)** Density distribution of Histone H3 expression of segmented cellular objects across cerebellum and hippocampal tissue samples. Dotted line indicates cutoff value of 0.25 for defining nucleated cellular objects.

Supplementary Figure 2

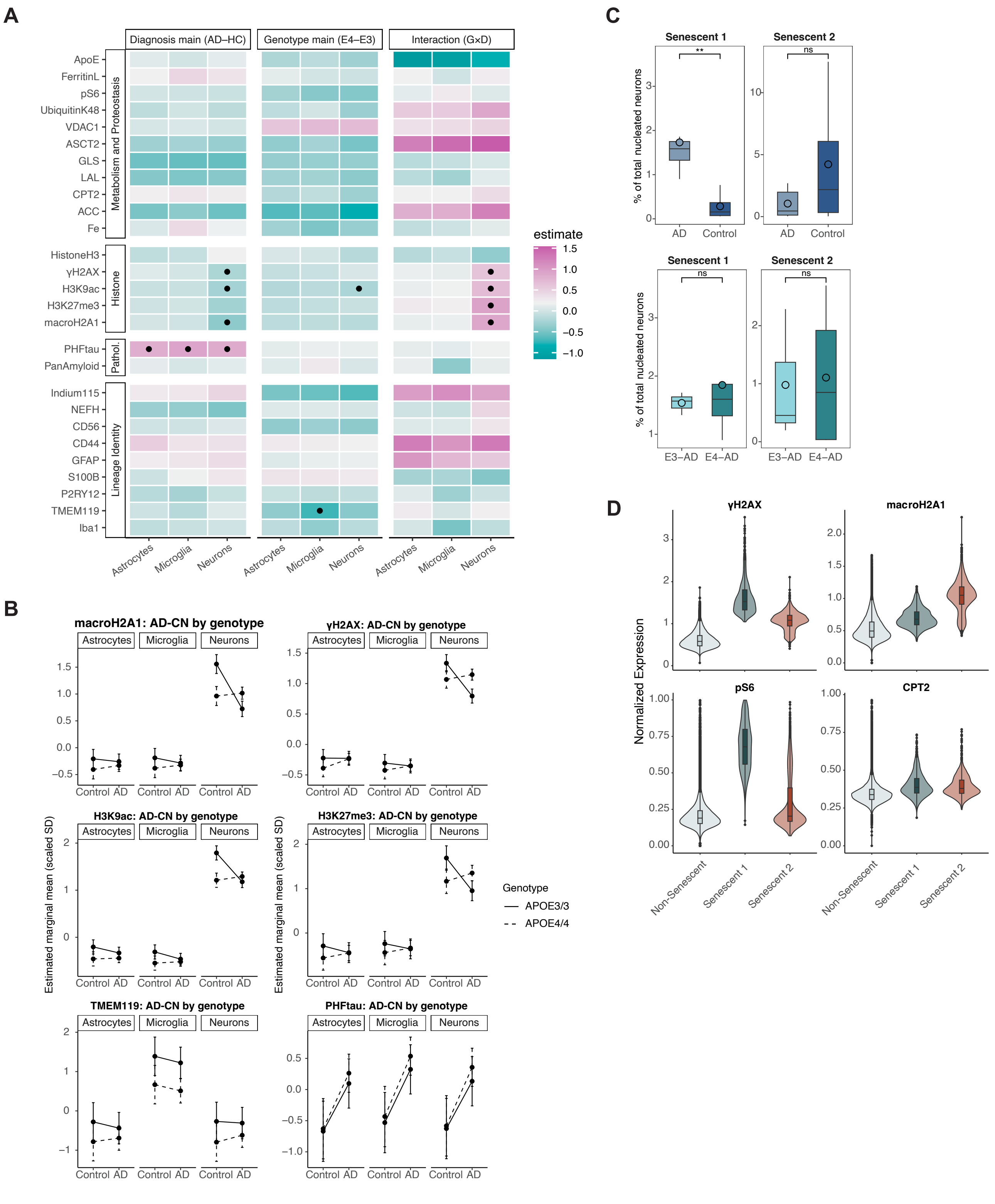

(A) Signed effect-size heatmaps. Tiles display signed estimates (SD units) for the genotype main effect (E4-E3, averaged over diagnosis), the diagnosis main effect (AD-CN, averaged over genotype), and the G×D interaction defined as (E4,AD-E4,CN)-(E3,AD-E3,CN). Dots denote effects meeting both statistical significance (FDR < 0.05) and the 0.2 SD relevance threshold. **(B)** Interaction plot of estimated marginal means from the linear mixed-effects model. Line type denotes genotype; points are model-based means with 95% confidence intervals. Non-parallel lines indicate a genotype-diagnosis interaction; vertical separation between the line

types at a given diagnosis reflects the genotype main effect; overall vertical shift from CN to AD reflects the diagnosis main effect. **(C)** Senescent 1 was found to be significantly enriched in AD neurons, implicating this population in disease-associated senescence, although there was no significant difference in abundance between E3-AD and E4-AD. **(D)** Senescent 1 was found to show elevated levels of γH2A.X and reduced macroH2A1 positivity relative to Senescent 2.

Supplementary Figure 3

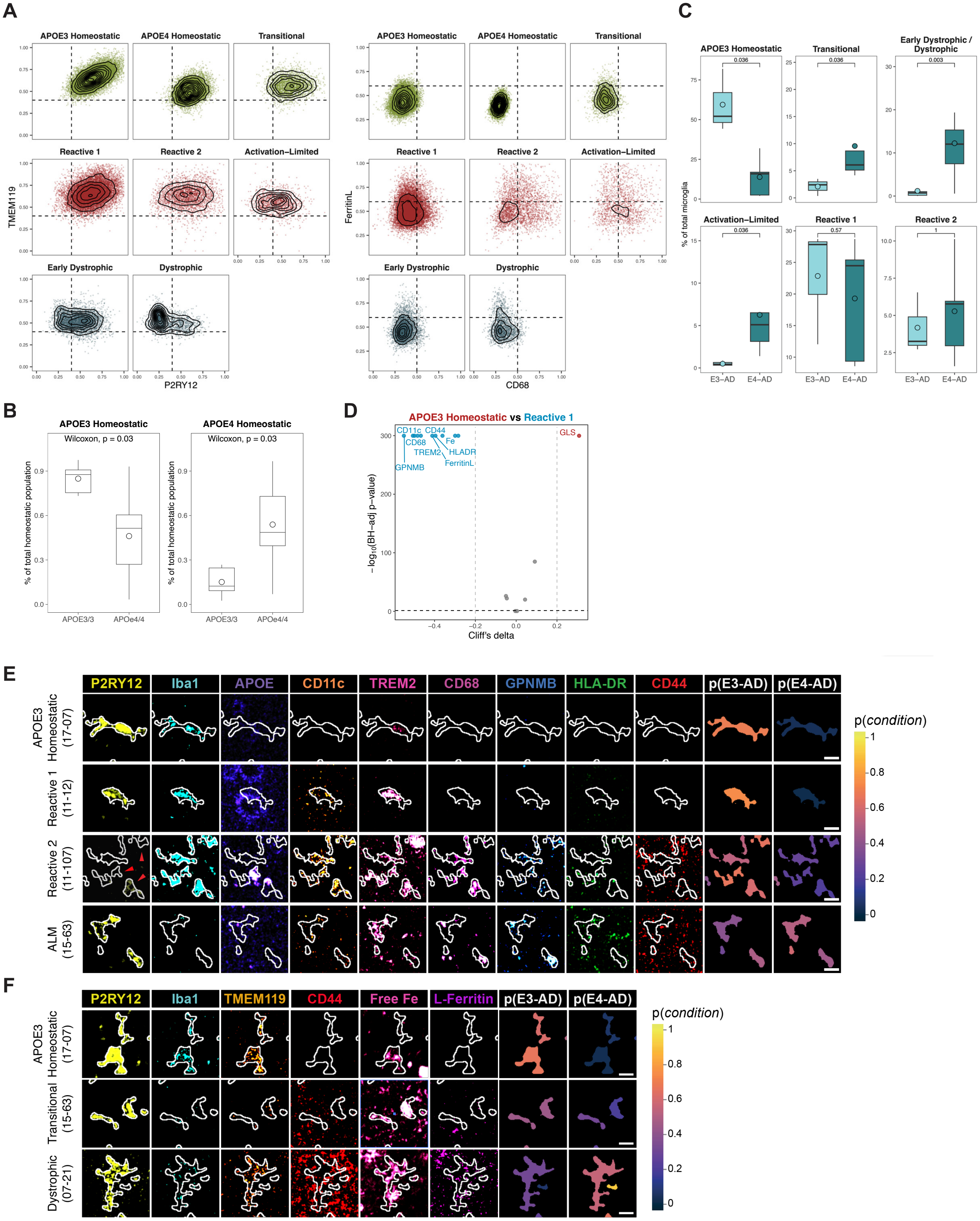

(A) Biaxial scatter plots of TMEM119 vs P2RY12, faceted by cluster (left). Clusters in the non-activated group (top row) show high expression of both markers. Reactive clusters (middle row) show partial reduction, and

dystrophic clusters (bottom row) exhibit pronounced loss, supporting the phenotypic group classification. Biaxial scatter plots of L-ferritin vs CD68, faceted by cluster (right). Clusters in the non-activated group show low expression of both markers, reactive clusters show elevated CD68 with emerging L-ferritin, and dystrophic clusters exhibit high L-ferritin, supporting the phenotypic group classification. **(B)** Frequencies of APOE3- vs APOE4-associated homeostatic clusters per individual, expressed as a percentage of the total homeostatic population. APOE3 Homeostatic cells are enriched in APOE3/3 individuals, whereas APOE4 Homeostatic cells are enriched in APOE4/4 individuals (Wilcoxon Rank-Sum $p < 0.05$). **(C)** Boxplots of cluster frequencies show that while Reactive 1 and Reactive 2 do not differ between genotypes, ALMs are specifically enriched in E4-AD individuals. E4-AD individuals show depletion of canonical homeostatic states in concert with an increase in APOE4-specific transitional and dystrophic states. **(D)** Volcano plot showing differential expression of key microglial activation markers between APOE3 Homeostatic and Reactive 1. **(E)** Representative multiplexed images of Reactive 1, Reactive 2 and ALMs. **(F)** Representative multiplexed images of Transitional and Dystrophic cells.

Supplementary Figure 4

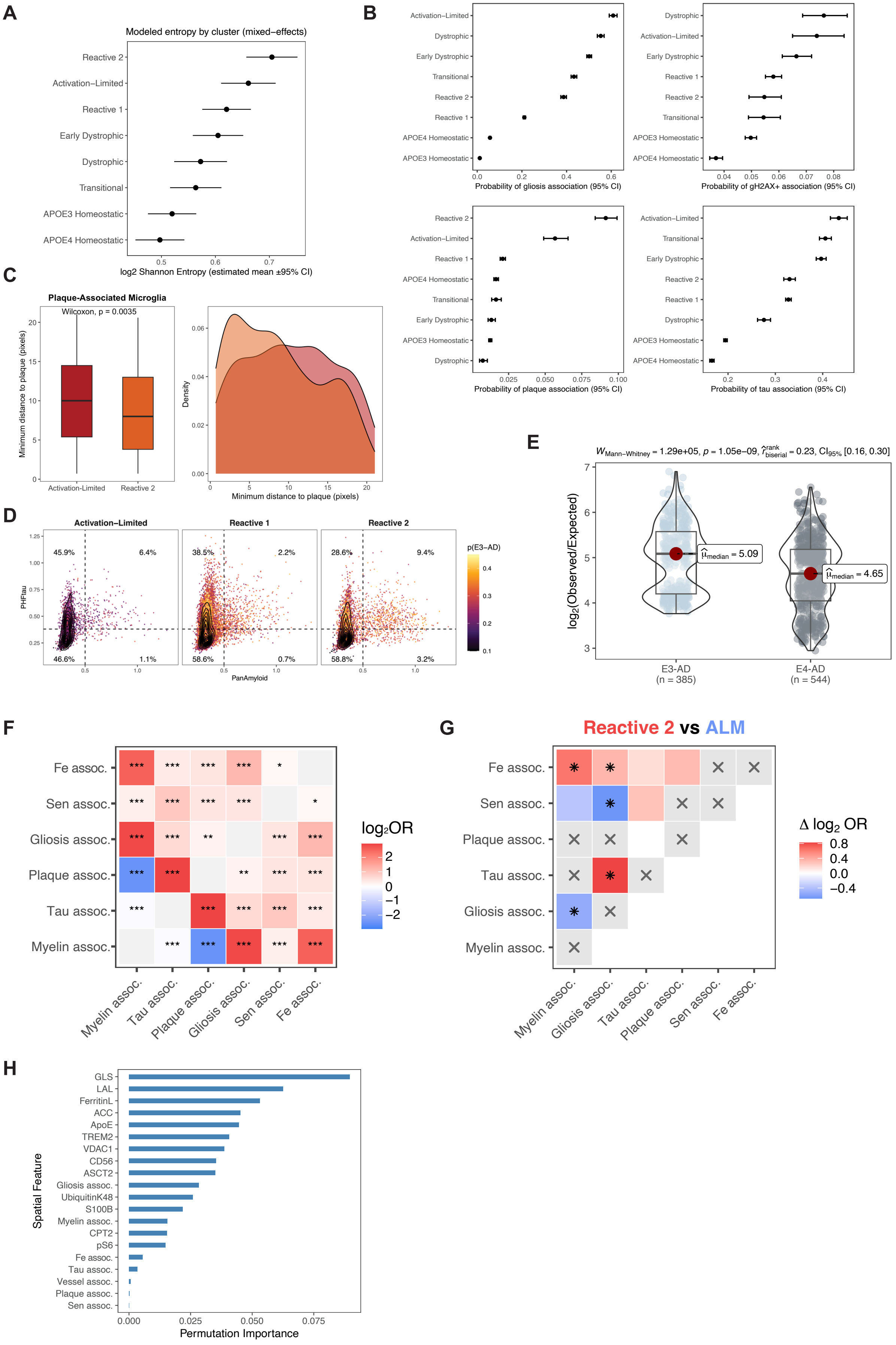

(A) Log_2 Shannon entropy per cell was modeled using a linear mixed-effects model with cluster as a fixed effect and donor as a random intercept. Points show estimated marginal means (±95% CI), adjusted for sample-level random effects. Clusters are ordered by estimated mean entropy. **(B)** Logistic regression odds ratios with 95% confidence intervals for spatial feature association by cluster. ALMs were more likely to be tau-associated relative to Reactive 2 microglia, whereas Reactive 2 microglia were significantly enriched for plaque association. Senescent (gH2AX+) neighbors were strongly associated with dystrophic and ALM states, indicating cluster-specific spatial pathology. **(C)** Spatial distance-to-plaque distributions. Reactive 2 microglia are significantly closer to amyloid plaques, consistent with plaque-associated DAM identity; ALMs remain farther away, reflecting non-plaque localization. **(D)** Spatial neighborhood analysis of DAMs versus ALMs. Points represent the mean pixel expression profile in a custom 20-pixel expansion area around individual microglia. Reactive 2 microglia preferentially localize near amyloid plaques (12.6%), consistent with plaque-associated DAM identity and a role in focal neuroinflammation. On the other hand, only 7.5% of ALMs are plaque-associated, underscoring divergent microenvironmental contexts. **(E)** Microglia in E3-AD are more spatially concentrated around plaques than in E4-AD. To quantify microglial coverage around plaques, we calculated the expected area of microglia in the expansion region assuming uniform distribution across the field of view (FOV). We then compared this to the observed microglial area within each plaque's 50-pixel expansion zone. The resulting enrichment ratio – defined as the log2 ratio of observed to expected microglial area coverage – served as a measure of microglial recruitment to plaques. Positive values indicate local enrichment, while negative values reflect under-representative relative to a uniform spatial distribution. This metric allows for direct comparison across clusters or plaque types while accounting for baseline microglial density in the tissue. **(F)** Global co-occurrence heatmap of spatial features. Each cell shows the log2 odds ratio for pairwise co-occurrence across all microglia (Fisher's exact test); positive values indicate enrichment; negative values indicate mutual exclusivity. Non-significant pairs (FDR ≥ 0.05) are muted. **(G)** Heatmap of differential co-occurrence between Reactive 2 and ALM clusters, showing $\Delta log_2 OR$ for each spatial feature pair. A 2×2×2 log-linear model tested whether the strength of association between each feature pair differed by genotype. Stars denote FDR-adjusted significance. **(H)** Feature permutation importance scores from a random forest classifier predicting microglial cluster identity from spatial features. Among the top-ranked predictors were pixel-level metabolic markers (e.g., GLS, LAL, APOE, VDAC1, TREM2) extracted from the neighborhood expansion region, underscoring the contribution of local metabolic tone to microglial state.

Supplementary Figure 5

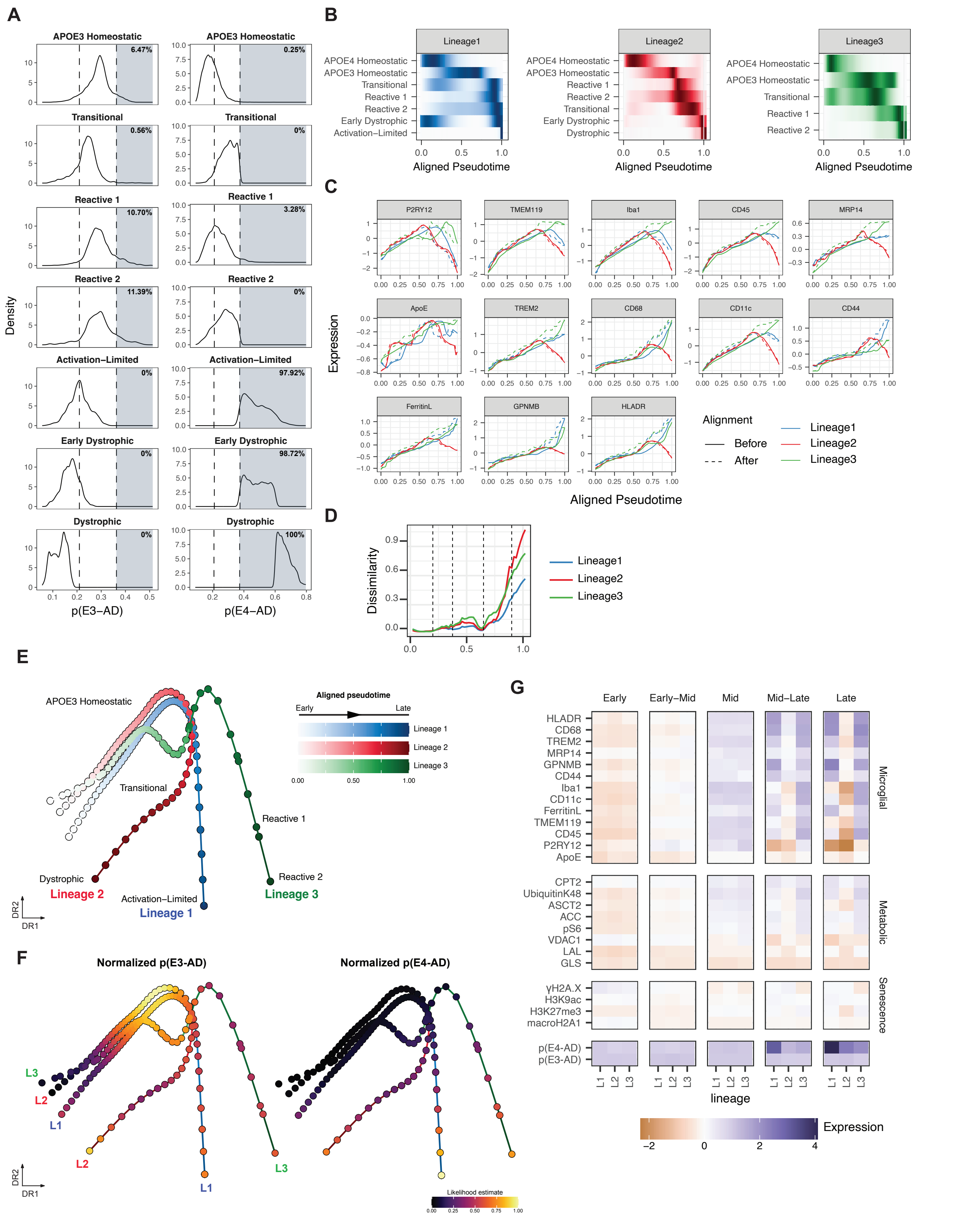

(A) Density distribution along the *p*(E3-AD) and *p*(E4-AD) continua; dotted lines denote the boundaries for low, middle, and high likelihood bins. Thresholds were determined via Gaussian Mixture Modeling of each likelihood continuum with k = 3 components. Percentage of cells per cluster found in the high likelihood bin is denoted in

the top right corner or each plot. Likelihood distributions reveal a graded shift along the *p*(E4-AD) continuum: homeostatic clusters are confined to low likelihoods, Reactive 1 and Reactive 2 occupy intermediate ranges, and ALMs dominate the far-right tail, reflecting their strong and distinct alignment with E4-AD. Homeostatic cells localize to low likelihoods, while Transitional and Dystrophic states are increasingly enriched at high *p*(E4-AD). **(B)** Distribution of cellular states across aligned pseudotime in the three inferred lineages. Rows correspond to annotated cell clusters (as applicable per lineage). Color intensity reflects the relative enrichment (density) of cells from each cluster at a given pseudotime point within each lineage, with darker shades indicating higher density. **(C)** Comparison of marker expression across pseudotime for each lineage prior to- (solid line) and post-trajectory alignment (dotted line). **(D)** Mean cosine dissimilarity between feature expression profiles, computed for each lineage relative to the other two. Vertical dotted lines indicate manually defined pseudotime bins. **(E)** Lineage embeddings constructed from the mean expression of microglial features within pseudotime bins along each aligned trajectory. All three lineages share a similar early topology, before diverging into distinct terminal phenotypes. **(F)** Lineage embeddings colored by normalized MELD condition-associated likelihood scores for E3-AD (left) and E4-AD (right). Select pseudotime bins are labeled by their predominant cluster identity. **(G)** Expression heatmap of features across aligned pseudotime for all lineages, stratified by pseudotime bin.

Supplementary Figure 6

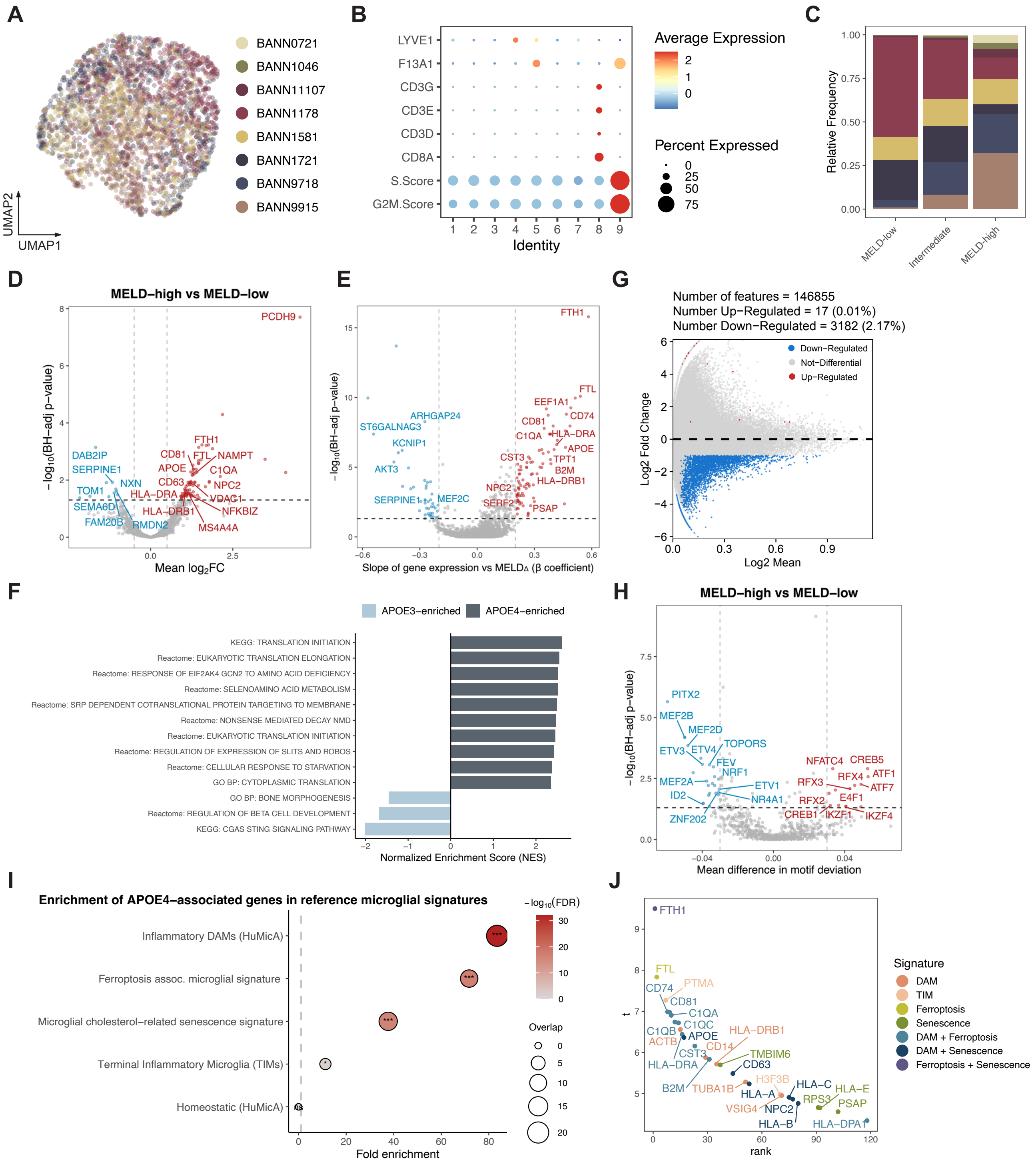

(A) UMAP colored by donor, demonstrating donor mixing following MultiVI integration. **(B)** Identification of cycling microglia and T cell populations. The balloon plot of Leiden clusters shows cluster 9 enriched for cycling cells (high G2M and S scores) and cluster 8 enriched for T cell markers (CD3D-G, CD8A). Clusters 8 and 9 were removed prior to MultiVI integration. **(C)** Donor contribution across $MELD_\Delta$ bins. **(D)** Differential gene expression between MELD-high and MELD-low cells. Pseudobulk counts were aggregated by donor and $MELD_\Delta$ bin prior to analysis with a limma-voom framework. Differentially expressed genes (FDR < 0.05, $\log_2$FC ≥ 0.5) are highlighted in red. **(E)** Continuous association analysis along $MELD_\Delta$. Each point represents a gene; coefficients indicate direction of association with the APOE continuum (positive: MELD-high; negative: MELD-low). Significant genes (FDR < 0.05 and |β| ≥ 0.2) are highlighted. **(F)** Genes were ranked by their moderated t-

statistics from donor-aware continuous $MELD_{\Delta}$ association analysis. The normalized enrichment score (NES) plot shows top 10 pathways enriched at each end of the continuum. Positive NES values indicate pathways upregulated in MELD-high (APOE4-associated) cells, while negative NES values indicate pathways enriched in MELD-low (APOE3-associated) cells. Significance was assessed using FDR-adjusted p-values. **(G)** Pairwise differential peak accessibility between MELD-high and MELD-low groups shown as an MA plot, illustrating effect size versus mean accessibility. **(H)** Differential transcription factor (TF) motif accessibility between MELD-high and MELD-low cells accessed using chromVAR motif deviation scores. The volcano plot shows mean deviation differences versus −log10(FDR), with significantly different motifs (FDR < 0.05, mean difference ≥ 0.03) highlighted in red. **(I)** Bubble plot showing enrichment of the APOE4-associated gene set across published microglial reference signatures. The x-axis indicates fold enrichment relative to expectation under the background gene universe, point size denotes the number of overlapping genes, and color denotes FDR-adjusted significance from one-sided Fisher's exact tests. **(J)** Genes from the APOE4 signature that overlap with a reference microglial signature are shown as individual points, colored by signature membership. The x-axis indicates gene rank based on GSEA ordering, and the y-axis indicates the moderated t-statistic from continuous association analysis as shown in (E), illustrating the relative positioning of inflammatory DAM, ferroptosis-, and senescence-associated genes along the APOE4-associated transcriptional axis.

Supplementary Figure 7

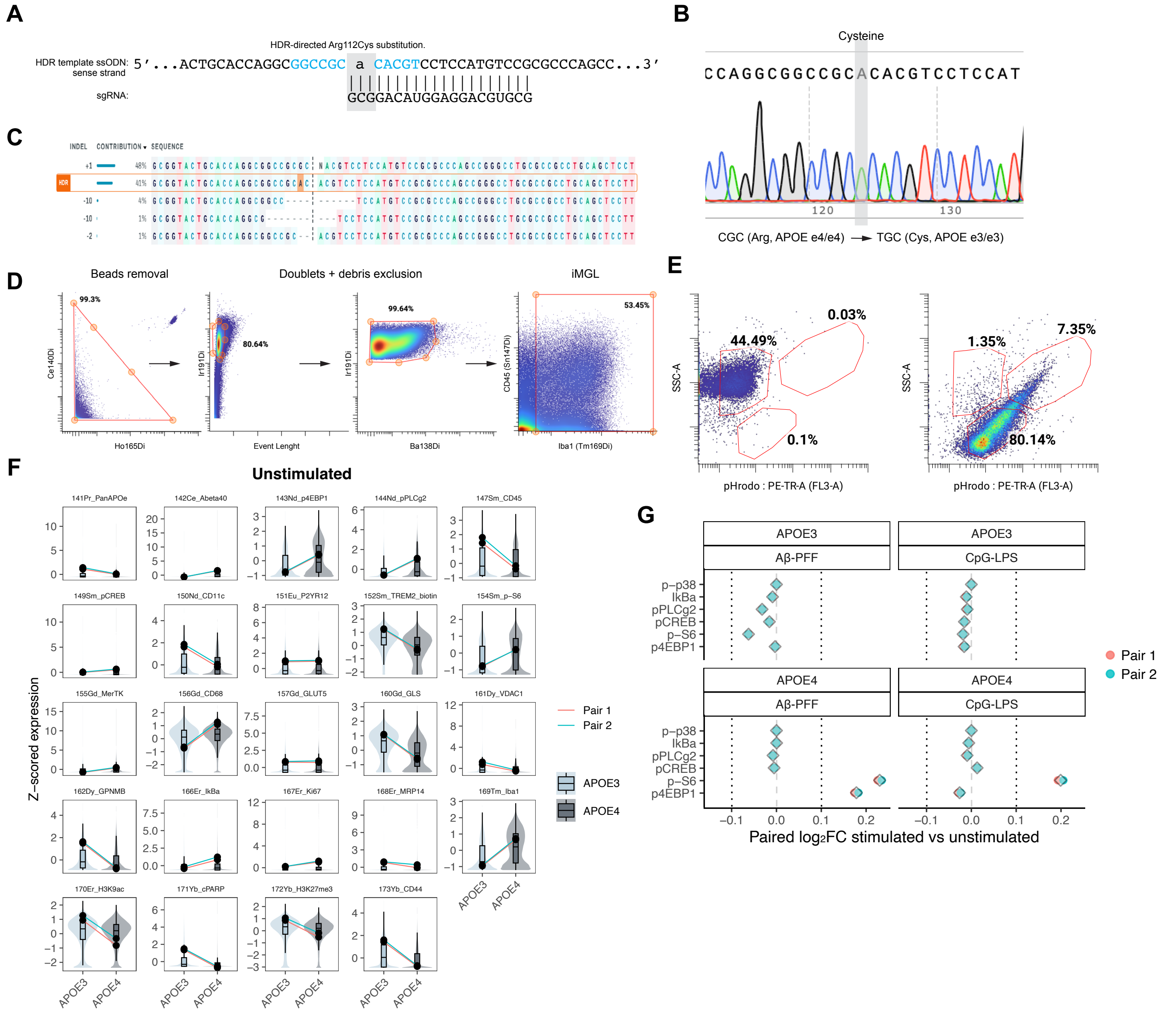

(A) Design of CRISPR/Cas9-mediated homology-directed repair (HDR) to introduce the APOE ε3 allele. The ssODN repair template encodes the Arg112Cys substitution (CGC→TGC), with the sgRNA targeting sequence shown aligned to the genomic locus. **(B)** Sanger sequencing chromatogram confirming successful editing at the APOE locus. The nucleotide substitution corresponding to Arg→Cys is highlighted, demonstrating conversion from APOE ε4/ε4 (CGC) to APOE ε3/ε3 (TGC). **(C)** Sequence decomposition analysis of edited populations showing HDR-mediated incorporation alongside indel profiles. The relative contribution of edited and indel-containing alleles is displayed, with the HDR sequence highlighted. **(D)** CyTOF gating workflow for identification of iMGLs. Sequential gating includes bead removal, exclusion of doublets and debris based on event length and DNA content, followed by identification of iMGLs based on CD45 and Iba1 expression. Percentages indicate the proportion of events retained at each step. **(E)** Flow cytometry gating strategy for the iMGL phagocytosis assay. Representative plots show gating of pHrodo-positive cells based on SSC-A versus pHrodo (PE-TR-A) fluorescence, with percentages indicating gated populations. **(F)** Comprehensive marker-level distributions of unstimulated microglia, stratified by APOE genotype. Violin and box plots display single-cell distributions for all measured markers across conditions and genotypes. Overlaid points indicate z-scored pseudobulked medians. Lines connect matched isogenic pairs, emphasizing consistency of observed genotype-driven differences across replicates. **(G)** Stimulus-induced responses by APOE genotype. Paired

log_2 fold-change (log_2FC) in marker expression following stimulation (Aβ/PFF or CpG/LPS, 12 min) relative to matched unstimulated controls, shown separately for APOE3 and APOE4 microglia. Each point represents the paired response for an individual isogenic pair; diamond-shaped points summarize the mean across pairs. Vertical dashed lines indicate no change (0) and reference thresholds (±0.1).

Supplementary Table 1

apoe_diag	AD_3	AD_3	AD_3	AD_4	AD_4	AD_4	AD_4	AD_4	control_3	control_3	control_4	control_4
CaseID	11-12	11-78	08-20	07-21	08-71	97-18	11-107	15-63	17-50	17-07	99-02	97-09
race	1	1	1	1	1	1	1	1	1	1	1	1
gender	2	1	1	2	1	2	1	2	1	2	2	2
expired_age	76	82	76	92	85	93	71	80	90	76	70	81
PMI	2	2.95	3.33	2.83	13	2.5	4.32	19	3.41	2.93	2	3
last_mmse_test_score		19	16	13	14		0	19	27	30		
last_mmse_interval_before_death		16	13	32	17		2	38	54	3		
Control	no	no	no	no	no	no	no	no	yes	yes	yes	yes
AD	yes	yes	yes	yes	yes	yes	yes	yes	no	no	no	no
PD	no	no	no	no	no	no	no	no	no	no	no	no
DLB	no	no	no	no	no	no	no	no	no	no	no	no
VAD	no	no	no	no	no	no	no	no	no	no	no	no
PSP	no	no	no	no	no	no	no	no	no	no	no	no
HS	no	no	no	no	no	no	no	no	no	no	no	no
DLDH	no	no	no	no	no	no	no	no	no	no	no	no
MND	no	no	no	no	no	no	no	no	no	no	no	no
CBD	no	no	no	no	no	no	no	no	no	no	no	no
PICKS	no	no	no	no	no	no	no	no	no	no	no	no
HD	no	no	no	no	no	no	no	no	no	no	no	no
MSA	no	no	no	no	no	no	no	no	no	no	no	no
ARG	no	no	no	no	no	no	no	no	no	no	no	no
CWMR	yes	yes	yes	yes	no	yes	yes	yes	no	yes	no	no
dementia_nos	no	no	no	no	no	no	no	no	no	no	no	no
MS	no	no	no	no	no	no	no	no	no	no	no	no
CAA	no	yes	yes	yes	yes	yes	yes	yes	no	yes	yes	yes
MCI	no	no	no	no	no	no	no	no	no	no	no	no
LBS	no	no	no	no	no	no	no	no	no	no	no	no
BRAIN_ABNORMAL	yes	no	no	no	no	no	no	no	no	yes	no	no
ACUTE_INFARCTS	no	no	no	no	no	no	no	no	no	no	no	no
ApoE	3/3	3/3	3/3	4/4	4/4	4/4	4/4	4/4	3/3	3/3	4/4	4/4
PlaqueF	3	3	3	3	3	3	3	3	0.5	0	1.5	2.5
PlaqueT	3	3	3	3	3	3	3	3	0	0	2	1.5
PlaqueP	3	3	3	3	3	2.5	3	3	0	0	2	1.5
PlaqueH	3	2.5	3	2.5	3	2.5	2.5	2	0	0	1	1
PlaqueE	3	3	3	3	3	3	2.5	3	0	0	1.75	1
PlaqueTotal	15	14.5	15	14.5	15	14	14	14	0.5	0	8.25	7.5
Plaque density	frequent	frequent	frequent	frequent	frequent	frequent	frequent	frequent	sparse	zero	sparse	sparse
TangleF	3	3	1.5	3	3	3	3	3	0	0	0	0
TangleT	3	3	3	3	3	3	3	3	0	0	0	0
TangleP	3	3	2	3	3	2.5	3	3	0	0.5	0	0
TangleE	3	3	3	3	3	3	3	3	1	1.5	0	1
TangleH	3	3	3	3	3	3	3	3	0.5	1.5	0	2
TangleTotal	15	15	12.5	15	15	14.5	15	15	1.5	3.5	0	3
Cerad NP	definite AD	definite AD	definite AD	definite AD	definite AD	definite AD	definite AD	definite AD	Criteria not met	Criteria not met	not AD	not AD
Braak score	VI	V	V	VI	VI	VI	VI	VI	I	III	I	II
NIA-R	high	high	high	high	high	high	high	high	Criteria not met	Criteria not met	criteria not met	criteria not met
Unified LB Stage	0. No Lewy bodies	0. No Lewy bodies	0. No Lewy bodies	0. No Lewy bodies	0. No Lewy bodies		0. No Lewy bodies	0. No Lewy bodies	0. No Lewy bodies	0. No Lewy bodies	0. No Lewy bodies	0. No Lewy bodies
infarct_total_volume	0	0	0	1	0	6.4	0	0	0.1	0	0	0
infarct_cerebral_total_volume	0	0	0	1	0	5.7	0	0	0.1	0	0	0
obt	0	0	0	0	0		0	0	0	0	0	0
brain_stem_ix_x	0	0	0	0	0		0	0	0	0		0
brain_stem_lc	0	0	0	0	0		0	0	0	0	0	0
bf_amygdala	0	0	0	0	0		0	0	0	0	0	0
bf_nbm			0									
brain_stem_sn	0	0	0	0	0		0	0	0	0	0	0
bf_trans	0	0	0	0	0		0	0	0	0	0	0
bf_cing	0	0	0	0	0		0	0	0	0	0	0
nctx_temporal	0	0	0	0	0		0	0	0	0	0	0
nctx_frontal	0	0	0	0	0		0	0	0	0	0	0
sum_lb_density	0	0	0	0	0		0	0	0	0		0
nctx_parietal	0	0	0	0	0		0	0	0	0	0	0
PathDXSummary	Alzheimer's Disease; Cerebral white matter rarefaction	Alzheimer's disease; Cerebral white matter rarefaction	Alzheimer's disease	Alzheimer's disease; Old lacunar infarct, left putamen; Cerebral white matter rarefaction	Alzheimer's disease with severe amyloid angiopathy; Marked loss of Purkinje cells, cerebellar cortex	Alzheimer's disease; multiple, small old infarcts of cerebral and cerebellar cortex	Alzheimer's disease; Cerebral white matter rarefaction	Alzheimer's disease; Cerebral white matter rarefaction	Control; Microscopic changes of Alzheimer's disease, insufficient for diagnosis; Old microscopic infarct, left middle frontal gyrus	Extensive leptomeningeal carcinomatosis, brain and spinal cord (history of invasive ductal carcinoma, right breast); Cerebral white matter rarefaction; Neurofibrillary tangles, mesial temporal lobe; Amyloid angiopathy,	Control; Alzheimer type II astrocytosis, consistent with terminal hepatic failure	Control
adcc_status	NA	NA	NA	NA	Accepted	NA	NA	Accepted	NA	NA	NA	NA
neurological_dx	Alzheimer's disease	Alzheimer's disease	Alzheimer's disease	Alzheimer's disease	Alzheimer's disease	Alzheimer's disease	Alzheimer's disease	Alzheimer's disease	Control	Control	Control	Control
neurological_dx_years	15	8	3	8	6	3	10	11				
neurological_dx_age	61	74	73	84	79	90	61	69				
dementia_yn	1	1	1	1	1	1	1	1	0	0	0	0
dementia_years	15	8	3	8	6	3	10	4				
demential_age	61	74	73	84	79	90	61	76				

Supplementary Table 2

Antigen	Clone	Catalog	Source	Isotope	Element	Concentration (ug/ml)
8OHG	15A3	ab62623	Abcam	69	Ga	1
HH3	D1H2	4499BF	Cell Signaling Technology	89	Y	2
CD56	MRQ42	156R-9-OEM	Cell Marque	113	In	2
Pan APOE	D7I9N	13366BF	Cell Signaling Technology	141	Pr	1
Abeta40	BDI350	ab20068	Abcam	142	Nd	1
ASCT2	CAL33	ab237704	Abcam	143	Nd	1
ACC	C83B10	52923SF	Cell Signaling Technology	144	Nd	1
CPT2	EPR13626	ab231162	Abcam	145	Nd	1
Abeta42	12F4	805501	Biolegend	146	Nd	1
CD45	D9M8I	47937SF	Cell Signaling Technology	147	Sm	1
CD105	Poly	AF1097	R&D Systems	148	Nd	1
CD31	EP3095	ab226157	Abcam	148	Nd	1
GLS	EP7212	ab214802	Abcam	149	Sm	1
polyUbiquitin48	EP8589	ab221211	Abcam	150	Nd	0.5
P2RY12	Poly	NBP2-33870	Novus	151	Eu	2
Biotin*	1D4-C5	409002	Biolegend	152	Sm	1
CD11c	EP1347Y	ab216655	Abcam	153	Eu	1
p-S6	D57.2.2E	4858BF	Cell Signaling Technology	154	Sm	0.5
MerTK	Y323	ab176887	Abcam	155	Gd	1
CD68	D4B9C	76437BF	Cell Signaling Technology	156	Gd	0.5
GLUT5	E-2	sc-271055	Santa Cruz Bio	157	Gd	0.5
gH2A.X	Poly	NB100-384	Novus	158	Gd	1
NEFH	RMdO 20	2836BF	Cell Signaling Technology	159	Tb	0.5
macroH2A1.2	14G7	MABE61	Millipore Sigma	160	Gd	1
VDAC1	20B12AF2	ab14734	Abcam	161	Dy	1
GPNMB	Poly	AF2550	R&D Systems	162	Dy	1
L-ferritin	D-9	sc-74513	Santa Cruz Bio	163	Dy	1
TMEM119	CL8714	41134BF	Cell Signaling Technology	164	Dy	1
MCNPase	SMI91	836404	Biolegend	165	Ho	2
LAL	9G7F12	ab36597	Abcam	166	Er	1
HLA-DR	EPR3692	ab209968	Abcam	167	Er	0.5
MRP14	Poly	LS_B12844	LS Bio	168	Er	0.125
Iba1	EPR16588	ab220815	Abcam	169	Tm	1
H3K9ac	C5B11	9649BF	Cell Signaling Technology	170	Er	1
S100b	E7C3A	90393BF	Cell Signaling Technology	171	Yb	1
H3K27me3	C36B11	9733BF	Cell Signaling Technology	172	Yb	1
CD44	IM7	103002	Biolegend	173	Yb	2
GFAP	E4L7M	80788BF	Cell Signaling Technology	174	Yb	0.5
Pan-Abeta	4G8	800701	Biolegend	175	Lu	0.5
PHFTau	PHF1_Tau	PHF1	Peter Davis Lab	176	Yb	0.25

***Primary Antibody**						
TREM2 (biotinylated)	Poly	BAF1828	R&D Systems	NA	NA	2

Supplementary Table 3

caseID	n_cells	AD	ApoE	gender	age	BraakScore	PlaqueTotal	TangleTotal
11-107	1996	yes	4/4	1	71	VI	14	15
97-18	1107	yes	4/4	2	93	VI	14	14.5
07-21	464	yes	4/4	2	92	VI	14.5	15
11-78	1650	yes	3/3	1	82	V	14.5	15
10-46	1569	yes	3/3	1	86	VI	14	15
15-81	1329	yes	3/3	1	81	VI	14	14.5

Supplementary Table 4

Antigen	Clone	Isotope	Element	Concentration (ug/ml)
p21-Waf1/Cip1	12D1	113	In	4
PanAPOe	D7I9N	141	Pr	2
Abeta40	BDI350	142	Nd	1
p4EBP1	2855BF	143	Nd	4
p-PLCg2	K86-689.37	144	Nd	2
CD4	RPA-T4	145	Nd	2
Abeta42	12F4	146	Nd	1
CD45	D9M8I	147	Sm	1
CD83	HB15e	148	Nd	2
p-CREB	polyclonal	149	Sm	2
CD11c	Bu15	150	Nd	2
P2YR12	polyclonal	151	Eu	2
Biotin	1D4-C5	152	Sm	2
TREM2-BIOTIN	polyclonal	152	Sm	2
CD14	HCD14	153	Eu	2
p-S6	D57.2.2E	154	Sm	0.5
MerTK	Y323	155	Gd	1
CD68	D4B9C	156	Gd	2
GLUT5	E-2	157	Gd	0.5
gH2AX pSer139	polyclonal	158	Gd	1
GLS	EP7212	160	Gd	1
VDAC1	20B12AF2	161	Dy	1
GPNMB	Poly	162	Dy	1
CD56	NCAM16.2	163	Dy	1
TMEM119	CL8714	164	Dy	1
p-p38 (T180/Y182)	36/ p38	165	Ho	2
IkBa	L35A5	166	Er	1
ki67	ki67	167	Er	4
MRP14	Poly	168	Er	0.125
Iba1	EPR16588	169	Tm	1
H3K9ac	C5B11	170	Er	1
cPARP	Asp214	171	Yb	3
H3K27me3	C36B11	172	Yb	1
CD44	IM7	173	Yb	2
HLADR	L243	174	Yb	1
CD206	15-2	175	Lu	2

Supplementary Table 5

ezSegmenter segmentation parameters

mask_name	composite_add	composite_subtract	object_shape	sigma	thereshold	hole_size	min_size	max_size
amyloid_plaques	"Beta Amyloid 1-40", "Beta amyloid 1-42",	NA	blob	1	85	auto	1000	100000
tau	"PHF-tau"	NA	blob	1	90	auto	200	100000
microglia_arms	"P2RY12", "Iba1", "TMEM119"	NA	projection	1	95	auto	300	100000
astrocyte_arms	"S100B", "GFAP"	NA	projection	0.7	85	auto	250	50000
vessels	"CD31_CD105"	NA	projection	2	98	auto	1000	100000
neurons_soma	"Indium115"	"HistoneH3"	blob	1	84	auto	1000	100000
neurons_axons	"NEFH"	NA	projection	2	95	auto	400	100000

Mask merger parameters

first_merge	merge_masks_list	cell_mask_suffix	merge_operation	percent_overlap	output_mask_name
TRUE	["neurons_axons"]	"neurons_soma"	combine	1	"neurons_combined"
TRUE	["neurons_combined"]	"whole_cell"	combine	90	"neurons_combined_merged"
FALSE	["microglia_arms", "astrocyte_arms"]	"whole_cell"	remove_duplicates	20	"microglia_arms_merged", "astrocyte_arms_merged"

Parameter	Definition
composite_add	Channels to add to composite channel.
composite_subtract	Channels to subtract from composite channel.
object_shape	General shape of the object, can be either \`blob\` or \`projection\`
sigma	The standard deviation for the Gaussian kernel to blur the image. Default is 1.
threshold	The per-fov-percentile threshold value (integer) for image thresholding.
hole_size	For any area smaller than \`hole_size\`, those holes are closed. Otherwise leave a 'auto' to determine the hole size based on image dimensions.
min_size	The minimum number of pixels required in a segmented object.
max_size	The maximum number of pixels permitted in a segmented object.
first_merge	Denotes if this is the first merge performed with a given merger base
merge_masks_list	List of object masks to merge to the base (cell) image. Merger occurs in order of appearance.
cell_mask_suffix	Suffix name of the cell mask files used as merger base. Usually \`whole_cell\` for Mesmer output.
merge_operation	\`combine\` integrates the base object into the final merged object. \`remove_duplicates\` identifies base objects that fulfill the merger criteria and removes the base object from the pool of base objects.
percent_overlap	Minimum percent threshold of a cell's area overlapping with an object that is required for a base cell mask to be merged into an object mask.

Round 1 merge
Base input: mesmer nuclear output
Operation: \`combine\` with neurons
Output: (a) neurons_combined_merged.tiff (b) remaining_cells (unmerged mesmer outputs)

Round 2 merge
Base input: remaining_cells from round 1
Operation: \`remove_duplicates\` for microglia and astrocytes
Output: (a) microglia_arms_duplicates_removed.tiff (b) astrocyte_arms_duplicates_removed.tiff (c) remaining_cells (unmerged mesmer outputs)

Supplementary Table 6

Pathology and Neuronal Damage

Feature	*Description*	*Type*
tau_expansion_mean	Mean PHF-tau intensity in expansion zone (NFTs)	Pixel-level
amyloid_expansion_mean	Mean pan-amyloid intensity in expansion (plaques)	Pixel-level
tau_expansion_pos	Area positive for NFT (binary)	Object-level
amyloid_expansion_pos	Area positive for amyloid (binary)	Object-level

Metabolic Environment

Feature	*Description*	
ASCT2_expansion_mean	Glutamine transporter, metabolic activaiton zone	Pixel-level
GLS_expansion_mean	Glutaminase - supports metabolic rewiring	Pixel-level
ACC_expansion_mean	Fatty acid synthesis indicator	Pixel-level
LAL_expansion_mean	Lysosomal activity	Pixel-level
pS6_expansion_mean	mTOR pathway activation in environment	Pixel-level

Iron and Lysosome Biology

Feature	*Description*	
ferritin_expansion_mean	Iron buffering or storage in the neighborhood	Pixel-level
fe_expansion_mean	Free iron levels in the neighborhood	Pixel-level

Astrocyte and Glial Landscape

Feature	*Description*	
GFAP_expansion_mean	Reactive astrocyte load in expansion zone	Pixel-level
S100B_expansion_mean	Calcium-binding astrocyte marker	Pixel-level
CD44_expansion_mean	Upregulated in astrocyte-DAM crosstalk	Pixel-level
gliosis_index	Double postivity for GFAP and CD44 expansion expression	Object-level

Epigenetic and Stress Signals

Feature	*Description*	
pct_macroH2A1_pos	Presence of nucleated macroH2A1+ neighbor	Object-level
pct_gH2AX_pos	Presence of nucleated gH2A+ neighbor	Object-level
pct_H3K27me3_pos	Presence of nucleated H3K27me3+ neighbor	Object-level
pct_H3K9ac_pos	Presence of nucleated H3K9ac+ neighbor	Object-level
min_dist_to_gH2AX_pos	Distance to nearest gH2AX+ senescent cell	Object-level

Vascular/White Matter Proximity

Feature	*Description*	
CD31_CD105_expansion_mean	Endothelial markers	Pixel-level
CD56_expansion_mean	Seen on vasculature or neural adhesion sites	Pixel-level
MCNPase_expansion_pos	Area positive for MCNPase (binary)	Object-level

Neighborhood Composition

Feature	*Description*	
niche_entropy	Shannon entropy of neighbor types (log2)	Object-level
percent_tau_pos_neighbors	Percentage of neighbors that are tau+	Object-level
percent_amyloid_pos_neighbors	Percentage of neighbors that are amyloid+	Object-level
min_dist_to_tau	Distance to nearest segmented tau+ object (if available, including neuror	Object-level
min_dist_to_amyloid	Distance to nearest segmented amyloid object (if available)	Object-level
min_dist_to_vessel	Distance to nearest vessel object (if available)	Object-level

APOE4-associated signature

FTH1	FTL	EEF1A1	MT-ATP6	RPL19	MT-CO2	PTMA	CD74	CD81	C1QA	MT-CO3
C1QB	RPS2	C1QC	ACTB	HLA-DRA	APOE	TPT1	MT-CYB	RPL10	MT-ND3	CST3
RPL15	RPS3A	RPL13	MT-ND2	RPS18	CD14	RPL13A	B2M	MRC1	RBMS3	HLA-DRB1
RPL30	TMBIM6	MT-ND4	RPL35	MT-ND1	RPS19	MT-CO1	CD63	RPL36A	RPL41	RPL29
TUBA1B	HLA-A	RPL18A	RPL28	RPS27A	RPS14	RPLP1	RPS15	RPL18	RPL17	H3F3B
VSIG4	RPL7A	HLA-C	NPC2	RPL32	HLA-B	FAU	RPL8	SERF2	RPS3	HLA-E
RPLP2	RPL24	PSAP	RPL9	RPL6	TMSB4X	RPL11	RPL3	LINC00486	HLA-DPA1	GRID2
RPS8	SLC9A9	LINC01684	RPS6	RPL5	RPS20	USP53	TRPS1	RPS24	MERTK	
